# Elevated glycosylated hemoglobin levels and their interactive effects on hypertension risk in nondiabetic Chinese population: a cross-sectional survey

**DOI:** 10.1186/s12872-020-01501-5

**Published:** 2020-05-12

**Authors:** Jian Song, Nana Wei, Yingying Zhao, Yuhong Jiang, Xuesen Wu, Huaiquan Gao

**Affiliations:** 1 School of public health, Bengbu medical college, Bengbu, 233000 Anhui Province China; 2 Department of general medicine, The First Affiliated Hospital of Bengbu Medical College, Bengbu, 233004 Anhui Province China; 3 Bengbu health board, 568 Nanhu road, Bengbu, 233000 Anhui Province China

**Keywords:** Hypertension, HbA1c, Obesity, Interaction

## Abstract

**Background:**

Abnormal glucose metabolism has been suggested to be involved in the development of hypertension. This study investigated the effect of the association and potential interaction of glycosylated hemoglobin (HbA1c) and other factors on the risk of hypertension among Chinese nondiabetic adults.

**Methods:**

As a cross-sectional survey, the current work deployed a questionnaire survey, anthropometric tests, and biochemical measures for each of the eligible participants. The HbA1c levels were quantified and grouped by quartiles. Correlations between HbA1c and hypertension, isolated systolic hypertension (ISH), and isolated diastolic hypertension (IDH) risk were investigated by logistic analyses. For evaluating the interactive effects, the parameters of relative excess risk due to interaction (RERI), attributable proportion due to interaction (AP), and synergy index (SI) were calculated, respectively.

**Results:**

In the current study, 1462 nondiabetic subjects were enrolled. In total, the prevalence rates of hypertension, ISH and IDH were 22.4, 9.6 and 4.5%, respectively. When HbA1c levels were grouped by quartile, it was revealed that the prevalence rates of hypertension and ISH were substantially elevated across groups (P_for trend_ < 0.001). In the multivariable logistic regression analyses, in comparison with the first quartile of HbA1c, the normalized OR for hypertension risk was 1.90 (95% CI: 1.28–2.80) for the highest quartile. Also, the risk of ISH was significantly increased with HbA1c level in the highest quartile relative to in the bottom quartile (OR: 2.23,95% CI:1.47–3.71). However, no significant relationship between the HbA1c level and IDH risk was observed (OR: 1.78, 95% CI: 0.82–3.84). Eventually, it was demonstrated from the interactive effect analysis that HbA1c significantly interacted with abdominal obesity (RERI: 1.48, 95% CI: 0.38–2.58; AP: 0.37, 95% CI: 0.14–0.60 and SI: 1.96, 95% CI: 1.06–3.62) and family history of hypertension (AP: 0.37, 95% CI: 0.05–0.70) in influencing the risk of hypertension in nondiabetic participants.

**Conclusion:**

Higher HbA1c levels significantly enhanced the risk of hypertension and ISH, but not IDH among Chinese nondiabetic adults. Moreover, the risk of hypertension was also aggravated by the upregulated HbA1c in a synergistic manner alongside abdominal obesity and family history of hypertension.

As one of the most common cardiovascular diseases, hypertension has become a prominent social public health problem globally [1]. A national survey of 451,755 participants from 31 provinces in China estimated that nearly 244.5 million people were suffering from hypertension [2]. What was worse, low rates of awareness, treatment, and control of hypertension were reported [2, 3]. Additionally, several meta-analyses have proved that hypertension was dramatically associated with the increased risk of a series of diseases such as Parkinson’s disease, stroke, and cancer [4–6]. It was believed that 54% of stroke cases and 47% of ischemic disease cases were attributable to hypertension worldwide [7]. Consequently, distinctly investigating the risk factors and effective predictors of hypertension are essential for alleviating the public health burden.

Often accompanied by abnormal glucose metabolism, hypertension may be observable in greater than two-thirds of patients suffering from type 2 diabetes [8]. It was suggested that the development of hypertension is consistent with hyperglycemia. Insulin resistance (IR), hyperinsulinemia, and the excitatory effects of hyperglycemia itself may be the underlying mechanisms of hypertension [8]. HbA1c, as a stable indicator of long-term glycemia, can precisely reflect the stability of glycemic control within nearly 8–12 weeks and was also correlated with defects of pancreatic β-cell function, as well as the degree of IR [9, 10]. Other evidence has increasingly demonstrated that not only patients with a higher risk of developing diabetes but also those with cardiovascular diseases could be identified by HbA1c level [11, 12]. Research has reported a significant relationship exists between a high-normal HbA1c level and an increased risk of arterial stiffness in individuals without type 2 diabetes [13]. To date, a few studies have analyzed the relationship between HbA1c and the risk of hypertension, and the conclusions were inconsistent. Additionally, ISH and IDH, as two subtypes of hypertension, were shown to have different pathophysiological mechanisms and distinctive risk factors [14, 15]. However, the relationship between HbA1c level and different types of hypertension remained unclear. Furthermore, as a multifactorial disease, hypertension was affected by a series of factors related to its occurrence and development. For instance, a remarkable interaction between smoking and overweightness has an impact on hypertension risk [16]. A case-control study in the Chinese population indicated that body mass index (BMI) dramatically interacted with a family history of hypertension to influence the risk of hypertension [17]. Conclusively, we hypothesized that HbA1c may have an effect in combination with other factors on the risk of hypertension.

Using data from the present cross-sectional survey, we aimed at: (1) investigating the association between HbA1c and risk of hypertension, ISH, IDH, respectively. (2) exploring potentially interactive effects of HbA1c together with other factors on the risk of hypertension.

## Methods

### Subjects

The participants in this study were recruited from a project labeled as an initiative aimed at “creating a provincial demonstration area of chronic disease management in the community,” which was conducted in Longzihu, Bengbu, China in 2015 and mainly designed to identify the epidemiological characteristics of chronic non-communicable diseases among the local residents. Multistage random sampling was utilized to select qualified subjects. The exclusion criteria included: (1) unable to complete the survey independently, (2) having a previous diagnosis of psychosis; or (3) temporary residents. In this study, for the purpose of analyzing the data of nondiabetics, those patients with diagnosed diabetes, a fasting plasma glucose (FPG) value of 7.0 mmol/L or higher, or receiving treatment for hyperglycemia were also excluded [18]. Eventually, 1462 nondiabetic subjects were selected. All of the participants were required to complete the whole survey in community clinics and each signed an informed consent form. The study protocol was approved by the ethics committee of Bengbu Medical College.

### Data collection

The data of general characteristics and lifestyle information were gathered by qualified investigators based on a face-to-face questionnaire survey. The established questionnaire included information about birth date, gender, smoking status, marital status (“currently married” or “currently not married”), educational level (“middle school graduate or lower” or “high school graduate or above”), income (“≤ 2,000 yuan” or “> 2000 yuan”), self-reported disease history, and family history of hypertension (yes or no). A positive family history of hypertension was defined as at least one parent or sibling with hypertension.

Blood pressure (BP) was measured based on unified standardized measurement methods [19]. Each subject was required to take a rest at least for 10 min in a quiet room prior to undergoing triple measurements of BP. The average results were calculated and adopted. If the difference was greater than 5 mmHg, the subject was required to undergo measurement of the BP again after a rest for at least 10 min. Hypertension was defined as when the systolic BP (SBP) was greater than or equal to 140 mmHg or diastolic BP (DBP) was greater or equal to 90 mmHg or the patient was using hypertension medications [20]. Individuals with an SBP of 140 mmHg or more and DBP of less than 90 mmHg were defined as having ISH, while IDH was defined as when the SBP was less than 140 mmHg and DBP was 90 mmHg or more [20]. The height and weight of each participant were measured with light indoor clothing. BMI was calculated as weight (kg)/height^2^ (m^2^). Participants with BMI values of 28 kg/m^2^ or more were regarded as showing general obesity [21]. For the measurement of waist circumference (WC), subjects were required to maintain a fasting state and an upright position. Abdominal obesity in males and females were defined as a WC of 90 cm or more or 85 cm or more, respectively [22]. Venous blood samples were collected in the morning and all the participants were required to complete overnight fasting for more than 8 h. Subsequently, HbA1c, FPG, and triglycerides (TG) were analyzed.

### Statistical methods

Statistical analyses were conducted using the R software. Normally distributed data were described as means± standard deviations (SD), which were further compared based on t-test. For non-normally distributed data, they were described as medians (P_25_, P_75_) and compared by the Wilcoxon rank-sum test. Additionally, percentages (%) were applied for the representation of categorical variables, which were subsequently analyzed using the chi-squared test. HbA1c levels could be classified into four quartiles (Q1-Q4). Univariate and multivariate logistic regression models were designed with odds ratios (ORs) and corresponding 95% confidence intervals (95% CIs). In order to obtain the best threshold of HbA1c to predict hypertension among nondiabetic subjects, receiver operating characteristic (ROC) curve analysis was adopted. Finally, three indicators reflecting interactive effects, including (1) the relative excess risk due to interaction (RERI), (2) attributable proportion due to interaction (AP), and (3) the synergy index (SI), were calculated. They were defined as follows: RERI = OR_11_ − OR_10_ − OR_01_ + 1, AP = RERI/ OR_11_, and SI = (OR_11_–1) / (OR_01_–1) + (OR_10_–1) [23, 24]. As an example, when we analyzed the interaction between HbA1c and general obesity, OR_11_ represented the effect of hypertension on individuals who had a increased level of HbA1c and BMI of 28 kg/m^2^ or greater; OR_10_ represented the effect of hypertension on individuals who had a higher HbA1c level and BMI of less than 28 kg/m^2^; and OR_01_ referred to the effect for hypertension whose had a lower HbA1c level and BMI of 28 kg/m^2^ or greater. Thus, OR_00_ referred to the effect for hypertension who had a lower HbA1c level and BMI of less than 28 kg/m^2^, which was regarded as the reference category. RERI = 0, AP = 0, or SI = 1 was considered as representing no additive interaction. Two-sided *p*-values were calculated and *p* < 0.05 was considered to be statistically significant.

## Results

Among the 1462 enrolled subjects, the mean age was 59.9 ± 11.3 years. Specifically, 692 males (40.5%) and 870 females (59.5%) were included, respectively. Overall, the rates of hypertension, ISH and IDH were 22.4, 9.6 and 4.5%, respectively. Males had a significantly higher rate of hypertension than females (*p* = 0.004). Compared with subjects with normotension (59.5 ± 11.4), those with hypertension (61.4 ± 10.8) exhibited a higher mean age (*p* = 0.008). Additionally, there was a remarkable difference in proportion of positive family history of hypertension (*p* = 0.042) between normotensive and hypertensive participants. Individuals with hypertension had a relatively higher smoking rate (32.1%) than did those with normotension (27.4%), without any statistically significant difference (*p* = 0.096). Meanwhile, no statistically significant differences in educational level (*p* = 0.068), marital status (*p* = 0.226), or income (*p* = 0.806) were observed. As for the obesity indices, both BMI (*p* < 0.001) and WC (*p* < 0.001) were proven to be dramatically upregulated in the hypertensive subjects relative to in the normotensive ones. Similarly, SBP (*p* < 0.001), DBP (*p* < 0.001), FPG (*p* = 0.037), TG (*p* < 0.001), and HbA1c (*p* = 0.002) varied markedly between the groups. The characteristics of enrolled subjects are described in detail in Table 1.

**Table 1:** Basic characteristic of the enrolled participants

Variables	Total (*N* = 1462)	Normtension (*N* = 1135)	Hypertension (*N* = 327)	*P* value
Gender				0.004^1^
Male(n(%))	692(40.5)	437 (38.5)	155 (47.4)	
Female (n(%))	870 (59.5)	698 (61.5)	172 (52.6)	
Mean age (SD)	59.9 (11.3)	59.5 (11.4)	61.4 (10.8)	0.008^2^
Educational level				0.068^1^
Middle school graduate or lower (n(%))	467 (31.9)	349 (30.7)	118 (36.1)	
High school graduate or higher(n(%))	995 (68.1)	786 (69.3)	209 (63.9)	
Marital status				0.226^1^
Currently married (n(%))	1234 (84.4)	951 (83.8)	283 (86.5)	
Currently not married (n(%))	228 (15.6)	184 (16.2)	44 (13.5)	
Income (yuan)				0.806^1^
< =2000 (n(%))	805 (55.1)	623 (54.9)	182 (55.7)	
> 2000 (n(%))	657 (44.9)	512 (45.1)	145 (44.3)	
Family history of hypertension(n(%))	266 (18.2)	194 (17.1)	72 (22.0)	0.042^1^
Smoking(%)	416 (28.5)	311 (27.4)	105 (32.1)	0.096^1^
BMI (kg/m^2^) (M(P_25_,P_75_))	24.3 (22.1,26.5)	23.8 (21.9,26.1)	25.5 (23.6,27.7)	< 0.001^3^
WC (cm) (M(P_25_,P_75_))	85.0 (80.0,91.0)	83.0 (78.0,90.0)	90.0 (83.0,96.0)	< 0.001^3^
SBP (mmHg) (M(P_25_,P_75_))	132 (125,140)	130 (120,135)	150 (145,160)	< 0.001^3^
DBP (mmHg) (M(P_25_,P_75_))	80 (72,85)	80 (70,83)	90 (80,100)	< 0.001^3^
FPG (mmol/L) (M(P_25_,P_75_))	4.9 (4.5,5.4)	4.8 (4.4,5.3)	4.9 (4.5,5.5)	0.037^3^
TG (mmol/L) (M(P_25_,P_75_))	1.4 (0.9,1.9)	1.3 (0.9,1.8)	1.6 (1.1,2.3)	< 0.001^3^
HbA1c(%) (M(P_25_,P_75_))	5.0 (4.4,5.6)	4.9 (4.4,5.5)	5.1 (4.6,5.8)	0.002^3^

^1^: Chi-squared test; ^2^:t-test; ^3^: Wilcoxon rank sum test

**Fig. 1:**
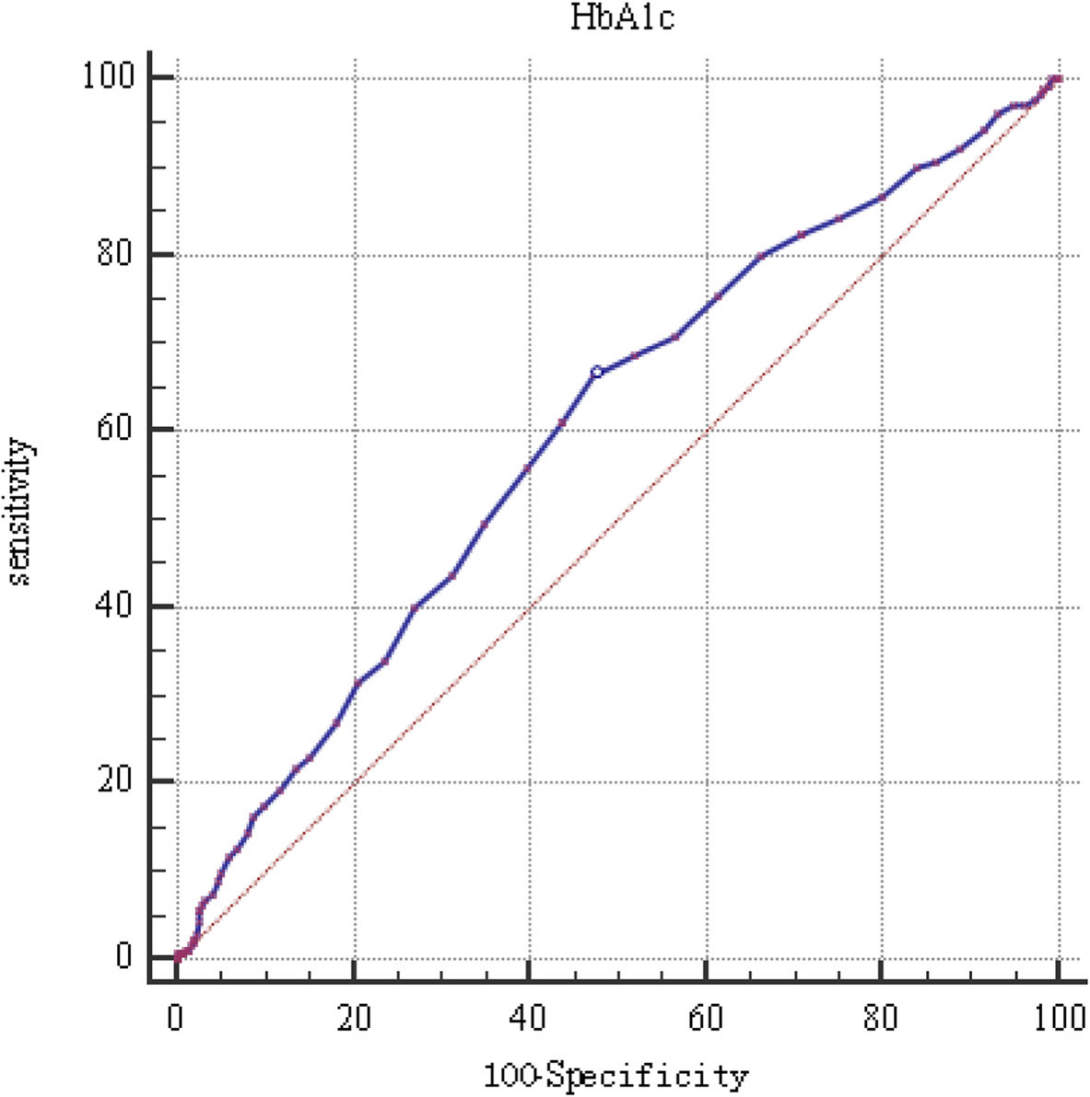
ROC curve analysis of HbA1c and hypertension risk in non-diabetic subjects

The results of HbA1c expression and the risks of hypertension, ISH, and IDH in nondiabetic subjects based on logistic regression analysis are listed in Table 2. The prevalence of hypertension was predominantly elevated with an increase in the quartile of HbA1c (*p*_for trend_ < 0.001). In the untreated model, there was a remarkable upregulation of the risk of hypertension across the quartiles of HbA1c, and the ORs (95% CI) were 1.00 (ref), 1.31 (0.90–1.92), 2.13 (1.48–3.06), and 2.57 (1.79–3.70), respectively. For the adjusted model, in contrast with the lowest HbA1c quartile, the OR (95%CI) was 1.90 (1.28–2.80) for the highest HbA1c quartile. Meanwhile, the risk of ISH was also significantly increased with HbA1c level in Q4 group than that in Q1 group (OR: 2.23, 95% CI: 1.47–3.71). The results also indicated that the per-unit increase in HbA1c would significantly enhance the risk of hypertension by 1.23-fold and the risk of ISH by 1.39-fold, respectively. However, no significant relationship between the HbA1c level and IDH risk was observed (OR: 1.78, 95% CI: 0.82–3.84). In Fig. 1, the ROC curve analysis suggested that the best threshold of HbA1c for predicting the risk of hypertension was 4.95%.

**Table 2:** HbA1c levels and risk of hypertension, ISH,IDH in non-diabetic population by logistic regression analysis

		Quartiles of HbA1c				p for trend
		Q1	Q2	Q3	Q4	
Hypertension	Unadjusted model	1.00(ref)	1.31 (0.90–1.92)	2.13 (1.48–3.06)	2.57 (1.79–3.70)	< 0.001
	Adjusted model ^1^	1.00(ref)	1.16 (0.78–1.74)	1.88 (1.28–2.75)	1.90 (1.28–2.80)	
	Continuous (per 1 SD)^1^	1.23 (1.08–1.40)				
	Unadjusted model	1.00(ref)	1.30 (0.69–2.43)	2.05 (1.15–3.65)	2.76 (1.57–4.84)	< 0.001
ISH	Adjusted model ^1^	1.00(ref)	1.28 (0.68–2.42)	1.87 (1.04–3.37)	2.23 (1.47–3.71)	
	Continuous (per 1 SD)^1^	1.39 (1.17–1.67)				
	Unadjusted model	1.00(ref)	1.48 (0.70–3.13)	1.74 (0.83–3.64)	1.80 (0.85–3.80)	0.106
IDH	Adjusted model ^1^	1.00(ref)	1.44 (0.68–3.10)	1.85 (0.87–3.90)	1.78 (0.82–3.84)	
	Continuous (per 1 SD)^1^	1.20 (0.94–1.53)				

^1^:adjusted for other variables;

As represented in Table 3, the participants were separated into four subgroups based on HbA1c level and other factors. After normalizing for confounders, the subjects with higher HbA1c level and a family history of hypertension simultaneously exhibited the highest OR (2.96, 95% CI: 1.90–4.62). It was estimated from AP (0.37, 95% CI: 0.05–0.70) that there was a combined effect of HbA1c and family history of hypertension on the risk of hypertension rather than from RERI (1.12, 95% CI: − 0.17–2.40) or SI (2.31, 95% CI: 0.83–6.44). Participants with alternative positive hypertension or general obesity showed a dramatically aggravated risk of hypertension in comparison with those with no positive HbA1c level and general obesity (OR: 2.37, 95% CI: 1.69–3.33 and OR: 1.79, 95% CI: 1.25–2.58, respectively). However, no noteworthy synergistic interaction was observed between HbA1c and general obesity (RERI: 1.12, 95% CI: − 0.18 to 2.42; AP: 0.24, 95% CI: − 0.01 to 0.49; and SI: 1.44, 95% CI: 0.91–2.27). Concerning abdominal obesity, the HbA1c (+) and abdominal obesity (+) subjects exhibited a higher risk of developing hypertension relative to the reference group (OR: 4.02, 95% CI: 2.81–5.74). There was a conspicuous additive interaction between the above parameters on hypertension (RERI: 1.48, 95% CI: 0.38–2.58; AP: 0.37, 95% CI: 0.14–0.60, and SI: 1.96, 95% CI: 1.06–3.62, respectively).

**Table 3:** interaction analysis of HbA1c with other factors on risk of hypertension

Variables		OR^1^(95%CI)	Measures of interaction^1^		
HbA1c	Family history of hypertension		RERI	AP	SI
–	–	1(ref)	1.12(−0.17–2.40)^2^	0.37 (0.05–0.70)^3^	2.31 (0.83–6.44)^2^
–	+	1.31 (0.80–2.13)			
+	–	1.54 (1.14–2.08)			
+	+	2.96 (1.90–4.62)			
HbA1c	General obesity				
–	–	1(ref)	1.12(−0.18–2.42)^2^	0.24(− 0.01–0.49)^2^	1.44 (0.91–2.27)^2^
–	+	1.98 (1.27–3.09)			
+	–	2.59 (1.71–3.92)			
+	+	4.69 (3.17–6.94)			
HbA1c	Abdominal obesity				
–	–	1(ref)	1.48 (0.38–2.58)^3^	0.37 (0.14–0.60)^3^	1.96 (1.06–3.62)^3^
–	+	2.10 (1.41–3.12)			
+	–	1.44 (0.92–2.22)			
+	+	4.02 (2.81–5.74)			

^1^:adjusted for other variables;

^2^:*p* > 0.05;

^3^:*p* < 0.05;

## Discussion

This population-based survey demonstrated that higher HbA1c levels dramatically aggravate the risk of hypertension in subjects without diabetes, further emphasizing the role of abnormal glucose metabolism in the pathogenesis of hypertension. Owing to specific merits, HbA1c generally serves as an effective indicator in the management of diabetes over FPG or postload plasma glucose. First, HbA1c has less biological variability and higher stability and, second, HbA1c could be less affected by relevant factors, such as acute infection, short-term lifestyle alterations, and recent eating behaviors [25]. Third, FPG only reflects the immediate glycemia level at the time of a single measurement; in contrast, HbA1c can stably indicate chronic glycemia levels, which reflect variations in average glycemia level across nearly two to 3 months. A cohort study consisting of 31,148 adults revealed that HbA1c was closely correlated with all-cause mortality and coronary heart disease in contrast to the fasting glucose [26]. Arbel et al. [27] investigated the relationship between glucometabolic markers (including admission glucose, FPG, and HbA1c) and the severity of coronary artery disease in nondiabetic patients, which indicated that only HbA1c was associated with the severity of coronary artery disease.

The relationship between glycemic control and hypertension can be explained by several possible mechanisms. First, function deficits of pancreatic β cells and IR could be indicated by the expression, of HbA1c [9, 10]. It was well-recognized that IR was the common pathophysiological foundation for the development of both type 2 diabetes and hypertension [28]. When the homeostasis model assessment of IR was applied to estimate IR, it was revealed that the result was dramatically upregulated across the quartile levels of HbA1c in Korean males without diabetes [29]. Additionally, HbA1c was also reported to be one of the best indices for identifying IR in obese nondiabetic individuals [10]. Second, numerous studies have implied that HbA1c may play a role in arterial stiffness via proinflammatory cell signaling and oxidative stress [30, 31]. It was demonstrated in a cross-sectional survey containing 11,014 Chinese participants that brachial–ankle pulse wave velocity and central SBP were markedly elevated across the quartiles of HbA1c [32]. Third, increased levels of HbAlc can contribute to endothelial damage that would further promote the release of endothelin from endothelial cells and inhibit the production of nitric oxide and prostacyclin, which would result in vasomotor dysfunction and further increase the BP [25, 33]. Moreover, it has been reported that there is a direct association between HbAlc and the activation of the renin–angiotensin– aldosterone-system [34]. Also, it has been indicated in clinical research that blood lipids could be positively regulated by the high level of HbAlc, which contributed to the increase in blood viscosity and furthered the incidence of cardiovascular diseases [25]. With the increase in HbA1c level, the number of cardiovascular risk factors clustering, including FPG, high total cholesterol, high TG, and high low-density lipoprotein cholesterol, was also dramatically upregulated [35].

To our knowledge, limited researches have investigated the influence of higher HbA1c levels on hypertension risk, and the conclusions were inconsistent. A cohort study of Americans including 9603 participants demonstrated that higher baseline HbA1c concentrations were predominantly associated with the incidence of hypertension independently of obesity indices and other factors in diabetic as well as nondiabetic individuals [36]. Similarly, in a women’s health study, 19,858 American women initially without diabetes were followed up with for a median of 11.6 years. The subjects were grouped based on HbA1c by clinical quintiles, and the hazard ratio (HR) for the highest HbA1c quintile in comparison with that of the lowest was statistically significant in both the univariable analysis and multivariable analysis [37]. However, when grouping HbA1c by quintiles, the above significant association was eliminated after normalizing for BMI. Moreover, elevated HbA1c levels dramatically aggravated the risk of hypertension in an independent manner even after normalizing traditional risk factors in general middle-aged and elderly Chinese subjects [38]. Besides that, the Framingham Heart Study demonstrated that high HbA1c expression was associated with the prevalence of hypertension, but it was only based on a univariate analysis [39]. In a Japanese cohort study with 5 years of follow-up, 9584 individuals were investigated, and elevated expressions of HbA1c were not associated with an increased risk of developing hypertension in the multivariable analysis [40]. An increment in HbA1c level was also reported not to be independently involved in the future development of hypertension among the Israeli population [41]. Kroke et al. [42] revealed that there was a nonsignificant relationship between HbA1c and arterial hypertension in nondiabetic participants; nevertheless, arterial hypertension was defined as a BP of 160/95 mmHg or greater. These inconsistencies may be explained by the diversity of HbA1c according to age, gender, and ethnicity.

Additionally, our results indicated a significant relationship between the HbA1c level and ISH risk but not IDH risk. Similarly, IR as indicated by TG–glucose index was suggested to be correlated with ISH risk rather than IDH risk [43]. In patients with type 2 diabetes, there was also an independent association between the duration of diabetes and ISH risk, suggesting that chronic hyperglycemia may play an essential role in the pathogenesis of ISH [44]. A cross-sectional investigation among middle-aged and elderly adults in China suggested that patients with ISH had a significantly higher prevalence of impaired glucose regulation and diabetes than those with IDH [45]. Overall, the prevalence of IDH was lower than that of ISH in hypertension subtypes. The distribution of hypertension subtypes was affected by various factors, like economic conditions, gender, and age [46]. As is known, ISH independently reflects arterial stiffness and is more common in the elderly, while IDH is independently related to an increase in arteriolar resistance and is more common in young and middle- aged people. However, it should be considered that most of the subjects enrolled in this survey were middle-aged and elderly adults, and the number of cases of IDH in this study was relatively small. Therefore, the association between HbA1c and IDH risk needs to be further explored, especially in young adults.

This study further demonstrated that HbAlc plays a significantly interactive role in the impact of abdominal obesity rather than general obesity on the risk of hypertension. Several studies have suggested that abdominal fat distribution may be more strongly related to adverse outcomes, such as cardiovascular diseases, than BMI. It is well acknowledged that obesity is a predominant risk factor of hypertension [47]. When evaluating the predicted performance outcomes of different obesity indices on hypertension, WC was superior to BMI based on ROC curve analysis [48]. It was illustrated that obesity was dramatically associated with elevated HbA1c levels in diabetic as well as nondiabetic subjects. Obesity can cause IR and result in poor glycemic control [25]. In addition, adipocytokines secreted from adipose tissue were involved in insulin resistance and beta cell dysfunction [49]. Furthermore, the occurrence of hypertension was a combinative consequence of genetic and environmental effects. A family history of hypertension was a simple and alternative genetic indicator. Moreover, a case-control study among Chinese individuals proved that a family history of hypertension and BMI had a positive impact on hypertension [17]. Our results also illustrated that HbAlc had a remarkable interaction with a family history of hypertension on the risk of hypertension.

There were several limitations in our study. First, the causality of the results failed to be inferred as this was a cross-sectional study. Second, the influences of various antihypertensive drugs on glucose metabolism varied but were not investigated in depth. However, it was shown that the effects of antihypertensive medication such as diuretics on HbA1c seemed to be of minor importance in diabetes as well as in nondiabetic individuals [50, 51]. Third, BP was measured in a single session and may be influenced by various external factors.

## Conclusion

This study demonstrated the independent and interactive effect of HbA1c on the risk of hypertension in nondiabetic Chinese subjects, suggesting that abnormal glucose metabolism has an essential role in the pathogenesis of hypertension. Further cohort studies with more research population are urgent to verify our results, and the underlying mechanism needs to be elucidated, which could eventually support more effective prevention strategies for hypertension.

## Data Availability

The datasets used and/or analyzed during the current study are available from the corresponding author on reasonable request.

## Abbreviations

ISH: Isolated systolic hypertension
IDH: Isolated diastolic hypertension
RERI: Relative excess risk due to interaction
AP: Attributable proportion due to interaction
SI: Synergy index
BMI: Body mass index
FPG: Fasting plasma glucose
BP: Blood pressure
SBP: Systolic BP
DBP: Diastolic BP
WC: Waist circumference
TG: Triglycerides
SD: Standard deviations
CIs: Confidence intervals
IR: Insulin resistance
